# Monitoring Phenolic Compounds in Rice during the Growing Season in Relation to Fungal and Mycotoxin Contamination

**DOI:** 10.3390/toxins12050341

**Published:** 2020-05-22

**Authors:** Paola Giorni, Silvia Rastelli, Sofia Fregonara, Terenzio Bertuzzi

**Affiliations:** 1 Department of Sustainable Crop Production—DIPROVES, Università Cattolica del Sacro Cuore, via Emilia Parmense 84, 29122 Piacenza, Italy; sofia.fregonara01@icatt.it; 2 Department of Animal, Food and Nutrition Science—DIANA, Università Cattolica del Sacro Cuore, Via Emilia Parmense 84, 29122 Piacenza, Italy; silvia.rastelli@unicatt.it (S.R.); terenzio.bertuzzi@unicatt.it (T.B.)

**Keywords:** rice, phenolic acids, fungi, mycotoxins, growing season

## Abstract

Total phenolic content (TPC) and several phenolic acids present in rice grains were compared with fungal infection and mycotoxin presence throughout the growing season. Samples of 4 rice varieties were collected in 2018 and 2019 at 3 different plant phenological stages. Total fungal and main mycotoxigenic fungi incidence were checked and mycotoxin content was analysed. On the same samples, TPC and the concentration of 8 main phenolic acids (chlorogenic acid, caffeic acid, syringic acid, 4-hydroxybenzoic acid (4-HBA), *p*-coumaric acid, ferulic acid, protocatecuic acid and gallic acid) were measured. The results showed significant differences between years for both fungal incidence and mycotoxin presence. In 2018 there was a lower fungal presence (42%) than in 2019 (57%) while, regarding mycotoxins, sterigmatocystin (STC) was found in almost all the samples and at all growing stages while deoxynivalenol (DON) was found particularly during ripening. An interesting relationship was found between fungal incidence and TPC, and some phenolic acids seemed to be more involved than others in the plant defense system. Ferulic acid and protocatecuic acid showed a different trend during the growing season depending on fungal incidence and resulted to be positively correlated with *p*-coumaric acid and 4-HBA that seem involved in mycotoxin containment in field.

## 1. Introduction

Rice (*Oryza sativa L.*) is one of the most important crops in the world [1]. It is mainly cultivated and consumed in Asian regions; its consumption is currently increasing all over the world [2]. The main rice producer in Europe is Italy, accounting for around 50% of total European production; rice cultivation is located principally in northern Italy (Piedmont, Lombardy and Veneto) [3].

Considerable quantitative and qualitative losses can occur in rice production from field to storage, due in particular to mould contamination [4,5]. Together with the most common diseases that affect Italian rice, such as panicle blast caused by *Pyricularia grisea* [6] and brown spot caused by *Bipolaris oryzae* [7], attention should be paid to mycotoxigenic fungal species found on paddy rice, which has been previously reported several times. All the principal mycotoxin producing genera, such as *Fusarium* spp., *Aspergillus* spp. and *Penicillium* spp., have been found on Italian paddy rice, but only deoxynivalenol (DON) produced by *F. graminearum*, aflatoxins (AFs) produced by *A. flavus* and sterigmatocystin (STC) produced by *A. versicolor* were recently reported as Italian paddy rice contaminants [8,9]. Mycotoxins can be produced by fungal secondary metabolisms when favorable environmental and substrate conditions occur during the growing season and their presence in grains can impact human health. In particular, STC has been considered by the International Agency for Research on Cancer (IARC) as a class 2B compound (possible human carcinogen).

Different studies have shown that the ability of vegetable species to resist the attack of microorganisms is often correlated with their content in phenolic compounds or polyphenols [10]. These compounds, widely distributed in plants [11], exhibit antifungal activity and can be produced by plants’ specialised metabolisms [10]. Thanks to a complex mechanism, plants produce an extremely diverse array of compounds that bestow metabolic plasticity essential for anticipating and responding to biotic and abiotic stresses [12]. In particular, phenolic acids are ubiquitous in plants and can be incorporated into the cell wall in response to biotic stress [13]. Reinforcement of the cell wall is generally accompanied by localized production of reactive oxygen species, driving cell wall cross linking, antimicrobial activity and defense signaling [14]. Moreover, from a nutritional and health point of view, phenolic compounds have demonstrated important nutraceutical properties; many studies have suggested that the intake of food rich in these compounds can reduce the risk of cardiovascular diseases and it seems they can have a role in protecting against type-2 diabetes and in counteracting obesity [15].

For their major role in the induction of resistance in plants and, as a consequence, their significant role against fungal infection, polyphenols have aroused a growing interest for a possible application in plant pathogens control strategies, even if limited knowledge is available on the effect of their concentrations on fungal populations in field [16,17].

Phenolic concentration in paddy rice is normally dependent on the cultivar, on the plant growth stage, on rice grain color and also on abiotic factors occurring during the growing season [18]. In particular, in rice grain there are present two groups of phenolic acids: derivates of hydroxybenzoic acids (such as gallic, p-hydroxybenzoic, salicylic, gentisic, protocatechuic, vanillic and syringic acids) and hydroxycinnamic acids (such as caffeic, *p*-coumaric, sinapic, ferulic and isoferulic acids), the first group being generally very low and the second one high in rice grain [1,19].

The aim of this study was to monitor the presence of fungal infection and mycotoxins in paddy rice during the growing season together with the polyphenol content in rice grains in order to define the plant response to fungal infection and the possible role of single polyphenols in the plant defense strategy against fungi.

## 2. Results and Discussion

### 2.1. Fungal Incidence and Mycotoxin Contamination

Significant differences (*p* ≤ 0.01) are found between the two years considered regarding the incidence of Genera with no mycotoxigenic capacity (other fungi) that resulted higher in 2019 than in the previous year, but no significant differences are found between the two years regarding the presence of mycotoxigenic fungi (*p* ≥ 0.05) (Table 1). Rice variety is relevant for fungal contamination, with Sole CL having the highest presence of fungi and CL26 the highest contamination with *Aspergillus* species (*p* ≤ 0.01) (Table 1). These data confirm results obtained in our previous studies where Sole CL was found to be the most contaminated rice variety and CL26 the most susceptible rice variety to *A. versicolor* infection [8,9].

No significant differences (*p* ≤ 0.01) are found between rice varieties for *Fusarium* spp. and *Penicillium* spp. contamination (Table 1). In particular, considering also the results of previous studies [8,9], *Penicillium* spp. seem to be found sporadically and generally in low amounts on Italian paddy rice in field. This is probably due to the weather conditions in the growing areas, characterized by very hot summers (mean daily temperature recorded from June to August was 25 °C in both considered years). The ecological needs of *Penicillium* spp. are usually different, showing maximum concentrations of spores at moderate temperature and high values of relative humidity [20].

Several studies have been conducted to verify possible rice variety resistance to *Fusarium* species especially against those associated with bakanae disease [21,22]; in this study no differences are found between rice varieties sampled, demonstrating their similar susceptibility to *Fusarium* spp. infection.

Considering the different stages of plant development, the reproductive phase, and in particular flowering, is considered the most susceptible to *Fusarium* species infection, especially in rainy springs that can favor spore dispersal [23]. Other fungi, like *Aspergillus* strains, can become more competitive in summers characterized by high temperatures and infection can be possible on grains throughout the ripening phase up to harvest time [24]. As already found in other studies on other cereals such as maize, fungal presence increased on grains during the growing season [24,25]; also in our study a significant increase in fungal incidence is noted from early dough stage and the highest incidence is registered at ripening (Table 1); only *Penicillium* spp. remains low and constant all season long (Table 1).

Contamination by STC is low and not statistically different for the two years considered (Table 1). The rice variety showing the highest contamination by STC is Sole CL (Table 1), confirming data also found in our previous studies where this variety seemed to be more susceptible to STC accumulation [8,9] even if it showed a low *Aspergillus* spp. incidence. This may be due to the fact that the level of mycotoxin content in samples might not be directly correlated to fungal incidence and *vice versa*, as already described by Schenck et al. [26].

**Figure 1:**
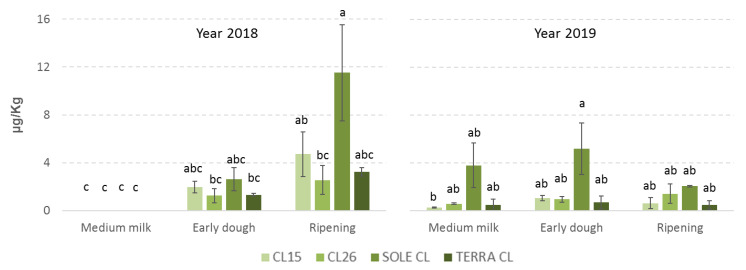
Mean contamination by sterigmatocystin (STC) of the 4 rice varieties considered in the study at the different sampling times observed in years 2018 and 2019. The error bars represent the standard deviations of the 3 replicates. Values with different letters differ significantly (*p* ≤ 0.05).

Sterigmatocystin accumulation in rice grains increases during the growing season with the highest point (*p* ≤ 0.01) at ripening (Table 1). Accumulation of mycotoxins during the growing season is often observed also in other cultures such as cereals [24,27], nuts [28] and grapes [29], however, regarding STC infection, even if no statistically significant differences are found between the years considered, it is possible to note interesting differences between accumulation in rice varieties. In particular, in 2018 STC infection was absent at medium milk for all the rice varieties considered and its increase was gradual up to ripening with only Sole CL showing a decisive boost from early dough to ripening resulting as almost five times higher (11.52 vs. 2.63) (Figure 1). In 2019, STC infection was present also at medium milk and it showed only a low increase for all the rice varieties, with the one exception of Sole CL that had considerably higher contamination than the other rice varieties already at medium milk, which slightly increased at early dough (+36%) and fell sharply at ripening (−60%) (Figure 1). This trend is observed in 2019 also for CL15 and Terra CL although at lower levels, while CL26 is the only rice variety that increased slowly but throughout the growing season (Figure 1).

**Figure 2:**
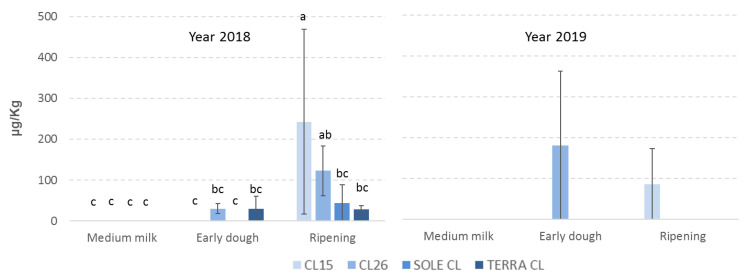
Mean contamination by deoxynivalenol (DON) of the 4 rice varieties considered in the study at the different sampling times observed in 2018 and 2019. The error bars represent the standard deviations of the 3 replicates. Values with different letters differ significantly (*p* ≤ 0.05).

Regarding DON, the highest contamination is observed in 2018 (*p* ≤ 0.05), however, for both years DON contamination can be considered very low being 200 µg/kg the legal limit in rice fixed by the European Commission (EU Regulation 1006/2015) (Table 1). It is likely that the meteorological conditions were particularly favorable for *F. graminearum* and *Fusarium* species in 2018 when mild temperatures (20 °C) and high rainfall (111 mm) occurred in May during flowering, which is considered the most susceptible phenological stage for Fusaria contamination [23], while in 2019 low temperatures and low rainfall recorded during the same period resulted in sporadic DON contamination (Table S1). Previous studies have demonstrated that areas with the lowest DON contamination are those with lower temperatures at the earing stage and flowering period [30]. All the rice varieties considered show a low contamination with no significant differences (*p* ≥ 0.05); however, DON contamination increases during the growing season with its highest point after early dough (*p* ≤ 0.01) (Table 1). In 2018 all the rice varieties had low levels of contamination at ripening, while in 2019 only two rice varieties showed DON contamination and at different plant growing stages (Figure 2). In particular, DON was found at early dough in CL26 and at ripening in CL15 (Figure 2), demonstrating the sporadic presence of this mycotoxin in Italian paddy rice.

### 2.2. Total Phenolic Content and Main Phenolic Composition

No significant differences are found in TPC between the four rice varieties considered in the study, while significant differences in TPC content are found for the years and sampling times (*p* ≤ 0.01) (Table 1).

Several studies have established that polyphenol concentration in whole rice grain is normally dependent on the cultivar, plant growth stage and abiotic factors, such as weather conditions [1,31]. Considering our results, differences between sampling times are quite normal and depend on the plant’s physiological necessities, while differences between the two years considered may be due to the varying meteorological conditions influencing the accumulation of phenolic compounds in plants. In particular, even if the temperatures were similar in both years, 2018 was characterized by intense rainfall, almost double that of 2019 (454 vs. 281 mm) (Table S1). However, phenolic compounds can be produced by the plant as a response to pathogenic fungal attack [32], and for this reason, differences during the growing season in our samples could also have been influenced by the different fungal infections observed at the different sampling times that could have significantly modified phenolic composition throughout the period.

**Figure 3:**
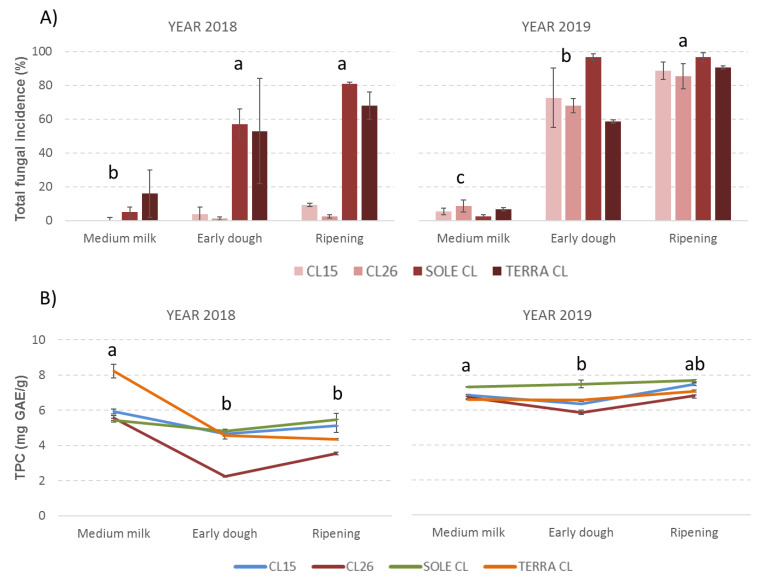
Mean incidence of total fungi infection (% of grains infected with fungi) (**A**) and mean total phenolic content (TPC) (**B**) found in the 4 rice varieties considered in the study at 3 different sampling times during the plant growing season observed in years 2018 and 2019. The error bars represent the standard deviations of the 3 replicates. Values with different letters differ significantly (*p* ≤ 0.05).

It is interesting to note that trends of TPC are similar for the varieties in both years considered although with different behavior (Figure 3). In 2018, TPC was highest at medium milk, it decreased widely at early dough and then increased again at ripening; in 2019 TPC had a different trend, remaining almost constant and at high levels throughout the growing season (Figure 3). In white rice, another previous study also underlined a higher TPC at the earlier plant growing stages (maximum at 1 week after flowering) than in the later stages [1].

However, taking into account the possible role of phenolic compounds in plant defense systems, it is quite interesting to compare TPC with total fungal infections registered by rice varieties during the years considered (Figure 3). In particular, at early dough, when fungal infection begins to be relevant, a general decrease in TPC is noted in both years. However, this reduction is higher in cases of lower fungal contamination (2018) and lower in cases of higher fungal contamination (2019). Regarding Sole CL, which has the highest fungal contamination in both the years considered, its TPC is the highest at ripening; at the same time, CL26, which has the lowest fungal contamination, has the lowest TPC at ripening (Figure 3). It may be that TPC is highest in the early plant development stages and decreases during the growing season; however, when fungal contamination becomes evident, TPC decrease is less evident probably because the plant needs a high TPC to protect itself or because the plant produces more polyphenols to fight fungal infection. This seems to confirm results obtained with sorghum where concentrations of phenolics were dependent on the cultivar, the stage of growth of the plant and also on the attack of pathogenic fungi [32].

Regarding phenolic acids present in rice grains, we took into consideration only those reported as predominant in rice plants and with a recognized role in the plant response system to pathogens [2].

Phenolic acid concentrations found in the four rice varieties considered in this study are reported in Table 2. All the phenolic acids considered show a considerable decrease in concentration from the stage of medium milk to the stage of early dough (falls of 2% to 97%); on the contrary, from early dough to ripening, a different behavior depending on the phenolic acid, which may continue to fall or may start to increase in concentration, is observed (Table 2). Conversely, ferulic acid is the only phenolic acid always found to be at higher concentrations at ripening than at early dough in both years for all the rice varieties considered (Table 2). Moreover, it almost always result higher at ripening than at medium milk, which is the growing stage where all the other phenolic acids considered reached their highest levels (Table 2). This could be related to its possible role in plant defense. In rice grains, ferulic acid is one of the most abundant [1,2] and is known to help protect the plant against pathogens and pests [33]. It is closely linked to cell wall structural components, such as cellulose, lignin and proteins [34] and its defensive role is in particular related to antioxidant activity associated with hydroxylation and methylation [2].

The same trend is observed also for protocatecuic acid, although not for all the rice varieties or for both years (Table 2). This phenolic acid is well known to help the rice plant to adsorb and utilize precipitated apoplasmic Fe from the root surface [35].

### 2.3. Correlations between Phenolic Acids, Fungal Contamination and Mycotoxin Content

When a Spearman’s correlation test is applied to the data set, significant correlations are found between some phenolic acids. In particular, ferulic acid results a positive correlation with 4-hydroxybenzoic acid (4-HBA), *p*-coumaric acid and protocatecuic acid, while it is negative relating to chlorogenic acid and gallic acid (Table 3). Moreover, *p*-coumaric acid is also positively correlated with 4-HBA (Table 3).

Significant correlations are also found between phenolic acids and fungal infection. Interestingly, *p*-coumaric acid results in a negative correlation with *Fusarium* spp., *Aspergillus* spp. and total fungi incidence, while protocatecuic acid is positively correlated with *Penicillium* spp. (Table 4). *Fusarium* species incidence is also negatively correlated with chlorogenic and 4-HBA and total fungal incidence is negatively correlated also with 4-HBA (Table 4). Regarding mycotoxins, both DON and STC are negatively correlated with *p*-coumaric acid and STC is also negatively correlated with 4-HBA (Table 4).

Considering the single years and the three sampling times, it is very interesting to note that during the first year, when fungal infection was lower, all the phenolic acids tested, with the exception of ferulic acid which is known to assist plant defense against pathogens [33], decreased during the growing season. In particular, it is important to underline a decrease from early dough stage to ripening that seems to determine a higher mycotoxin content. During the second year, when fungal infection was higher, both phenolic acids that correlate with the presence of fungal species (*p*-coumaric acid and 4-HBA) show an increase from early dough to ripening stage having as a final result a lower STC and DON content in the rice sampled.

This is quite interesting because *p*-coumaric acid and 4-HBA could have an important role in mycotoxin containment in field. Recently, different phenolic acids have demonstrated their great ability to reduce some mycotoxins in in vitro trials; in particular, ochratoxin A produced by *A. carbonarius* [36] or DON and T2-HT2 produced by *Fusarium* species [37].

## 3. Conclusions

This study has contributed to further understanding of the role of phenolic compounds in rice plant defense against fungal infection and for mycotoxin containment in field. In particular, total phenolic content (TPC) was strictly related to the plant development stage and fungal presence. Regarding individual phenolic acids, some among those considered showed quite interesting results. Ferulic acid at higher levels and protocatecuic acid showed different behavior from other phenolic acids, increasing especially when fungal presence begins to be consistent. Moreover, these two compounds were positively correlated with other phenolic acids (*p*-coumaric acid and 4-HBA) that seem to reduce mycotoxin infection. The specific effect of these molecules on mycotoxigenic fungi and different mycotoxins merits further study, and the possibility of selection of rice varieties that are richer in some phenolic compounds may become a new strategy to prevent mycotoxin contamination in field and consequently reduce food losses.

## 4. Materials and Methods

### 4.1. Field Samples

For two consecutive years (2018 and 2019), 4 rice varieties were cultivated in 2 experimental fields located close to Mortara (PV) in Lombardy, the main Italian rice production region. The rice varieties were long B grain (CL26) and round grain rice varieties (CL15, Sole CL and Terra CL).

Rice grain samples were undertaken at 3 different phenological stages: medium milk (BBCH 75), early dough (BBCH 83) and ripening (BBCH 89).

Plants of each rice variety were collected from 3 different plots (around 250 m^2^ each) with an X-shape design, each plot representing a replicate. The plants were then shelled and the grains obtained (around 500 g for each replicate) considered as representative.

Samples were used for mycological analyses and then dried, milled using a cyclone hammer mill (1 mm sieve, Pulverisette, Fritsch GmbH, Idar-Oberstein, Germany), homogenised and kept at 4 °C until mycotoxin and phenolic compound analysis.

### 4.2. Monitoring of Mycotoxigenic Fungi

Fifty kernels were randomly chosen from each sample, surface disinfected and transferred onto Petri dishes containing potato dextrose agar (PDA, Biolife, Milano, Italy). The Petri dishes were incubated at 25 °C (12 h light photoperiod) and after 5–7 days the incidence of kernels infected by fungi was quantified. As incidence, we used the percentage of grains infected by fungi of all the grains considered (50 grains for each replicate). *Fusarium* spp., *Aspergillus* spp. and *Penicillium* spp. isolates were identified at genus level thanks to observations by binocular microscope (×40). All the fungal strains isolated from rice and not belonging to these 3 mycotoxigenic genera were counted and reported as “other fungi”. The sum of all the fungal strains collected from samples, both belonging to mycotoxigenic genera and to other genera, were indicated as “total fungi”.

### 4.3. Monitoring of Mycotoxins

The analyses were carried out using the following methods: deoxynivalenol (DON) by GC-MS and sterigmatocystin (STC) by LC-MS/MS. The analyses were recently described in the work of Bertuzzi et al. [8]. Briefly, DON, after extraction and purification through a Trilogy-Puritox Trichothecenes column (R-Biopharm, Glasgow, UK), was derivatised with 200 µL of trimethilsilylimidazole-trimethilclorosilane (1 + 0.2 *v*/*v*) for 15 min in subdued light and extracted in hexane. The hexane phase was injected into GC-MS. GC-MS analysis was carried out using a TraceGQ Ultra coupled with ISQ single quadrupole mass spectrometry (Thermo-Fisher Scientific, San Jose, CA, USA). The analysis was carried out using a capillary column Rtx-5MS, 30 m × 0.25 mm i.d., 0.25 µm film thickness. Limit of detection (LOD) and limit of quantification (LOQ) were 5 and 15 µg kg^−1^, respectively; the average recovery was 92.4% ± 2.6%.

After extraction, purification through an immunoaffinity column (R-Biopharm, Glasgow, UK), and addition of isotopically labelled STC standard, STC was determined by LC-MS/MS system consisting of a LC 1.4 Surveyor pump, a Quantum Discovery Max triple-quadrupole mass spectrometer (Thermo-Fisher Scientific, San Jose, CA, USA) and a PAL 1.3.1 sampling system (CTC Analytics AG, Zwingen, Switzerland). STC was chromatographed on a Betasil RP-18 column (5 µm particle size, 150 × 2.1 mm, Thermo-Fisher) with a gradient acetonitrile-water (both acidified with 0.2% formic acid; flow rate 0.2 mL min^−1^); the ionization was performed using positive atmospheric pressure chemical ionization (APCI). The LOD and the LOQ were 0.05 and 0.15 µg kg^−1^, respectively. The average recovery was 90.4% ± 4.2%.

### 4.4. Determination of Total Phenolic Content (TPC)

Extracts were prepared by adding 4 mg of sample rice flour in 40 mL of methanol and maintaining under agitation for 1 h at room temperature. Centrifugation for 3 min at 5000 rpm was conducted after extraction to better separate supernatant.

Gallic acid was used as standard compound; concentrations of 40, 80, 120, 160, 200, 400 and 600 µg/mL of gallic acid were prepared in methanol and used to plot the calibration curve (y = 0.008x − 1.392, R^2^ = 0.992).

Total phenolic content (TPC) was determined by the Folin–Ciocalteu colorimetric method with minor modification [38]. Briefly, 1 mL of crude extracts or standard solutions were added to 5 mL of 10-fold diluted Folin–Ciocalteu reagent and then neutralized with 4 mL of saturated sodium carbonate (75 g/L). The resulting mixtures were incubated for 1 h at room temperature in darkness. The absorbance of the samples was measured at 765 nm spectrophotometrically (UV-1280, Shimadzu, Japan).

The total phenolic content was expressed as milligrams of gallic acid equivalent (mg GAE) per gram (g) of rice flour.

### 4.5. Determination of Phenolic Acids

The extracts prepared for TPC were diluted (1 + 4) with water: acetonitrile = 8 + 2 *v*/*v* and 20 µL was injected into an LC-MS/MS system. The HPLC-MS/MS system consisted of a LC 1.4 Surveyor pump (Thermo Fisher Scientific, San Jose, CA, USA), a PAL 1.3.1 sampling system (CTC Analytics AG, Zwingen, Switzerland) and a Quantum Discovery Max triple quadrupole mass spectrometer; the system was controlled by Excalibur 1.4 software (Thermo Fisher Scientific). Phenolic acids were chromatographed on a Betasil RP-18 column (5 µm particle size, 150 × 2.1 mm, Thermo-Fisher) and separated using gradient elution with water (A) and acetonitrile (B), both acidified with 0.2% formic acid. The gradient program was from 90% to 45% for solvent A within 9 min.; then, from 45% to 90% in 1 min and conditioning of the column for 7 min. The flow rate was 0.2 mL min^−1^. Ionisation was carried out with an ESI interface (Thermo-Fisher Scientific); for ferulic acid, *p*-coumaric acid, caffeic acid (hydroxycinnamic acids), syringic acid, chlorogenic acid and 4-hydroxybenzoic acid (4-HBA), ionisation was carried out in positive mode as follows: spray capillary voltage 4200 kV; sheath and auxiliary gas 35 and 14 psi, respectively; skimmer 6 V and temperature of the heated capillary 350 °C. Mass spectrometric analysis was performed in selected reaction monitoring (SRM). For fragmentation of the [M + H]^+^ ions, the argon collision pressure was set to 1.2 mTorr and the collision energy to 10 and 25 V. For gallic acid and protocatechuic acid, the ionisation was carried out in negative mode as follows with the spray capillary voltage fixed to 3500 kV. Mass spectrometric analysis was performed in selected reaction monitoring (SRM). For fragmentation of the [M - H]^−^ ions, the argon collision pressure was set to 1.2 mTorr and the collision energy to 16 and 30 V.

### 4.6. Data Analysis

The data were transformed before statistical analysis; in particular, fungal incidence was arcsine transformed and mycotoxin content was ln transformed [39]. Analysis of variance (ANOVA) was calculated using the statistical package IBM SPSS statistics 21 (IBM Corp., Armonk, NY, USA) while significant differences were highlighted using the Tukey test (*p* ≥ 95%) for mean separation. Data correlation was evaluated by Spearman’s correlation test (*p* ≥ 95%).

## Figures and Tables

**Table 1:** Analysis of variance (ANOVA) of fungal incidence (% of infected grains) and contamination of sterigmatocystin (STC) and deoxynivalenol (DON) at different sampling times in 4 different rice varieties. Data refer to mean data ± standard deviation; all experiments were conducted with three replicates. Different letters mean significant differences using the Tukey Test; N.S.: not significant; *: *p* ≤ 0.05; **: *p* ≤ 0.01.

**Factor A: Year**									
		**2018**		**2019**					
*Fusarium* spp. (%)	n.s.	9.73 ± 8.42		8.39 ± 7.07					
*Aspergillus* spp. (%)	n.s.	0.70 ± 2.00		0.72 ± 1.71					
*Penicillium* spp. (%)	n.s.	1.36 ± 4.28		0.78 ± 1.51					
Other fungi (%)	**	30.45 ± 32.70	B	46.94 ± 31.06	A				
STC (µg/kg)	n.s.	2.48 ± 3.50		1.47 ± 1.60					
DON (µg/kg)	*	38.35 ± 99.30	A	22.33 ± 72.94	B				
TPC (mg GAE/g)	**	2.725 ± 0.585	A	2.299 ± 0.191	B				
**Factor B: Rice Variety**									
		**CL15**		**CL26**		**SOLE CL**		**TERRA CL**	
*Fusarium* spp. (%)	n.s.	10.67 ± 9.57		5.38 ± 4.73		9.60 ± 7.77		9.47 ± 6.30	
*Aspergillus* spp. (%)	**	0.13 ± 0.50	B	2.10 ± 2.63	A	0.67 ± 2.02	B	0.13 ± 0.50	B
*Penicillium* spp. (%)	n.s.	0.93 ± 1.91		0.46 ± 0.84		0.40 ± 0.80		2.13 ± 5.08	
Other fungi (%)	*	38.00 ± 27.07	AB	38.31 ± 29.41	AB	48.27 ± 35.54	A	37.87 ± 30.13	B
STC (µg/kg)	**	1.44 ± 1.63	B	0.97 ± 0.77	B	4.20 ± 3.78	A	1.04 ± 1.02	B
DON (µg/kg)	n.s.	54.91 ± 121.45		51.58 ± 106.82		7.33 ± 21.95		9.75 ± 17.24	
TPC (mg GAE/g)	n.s.	2.426 ± 0.304		2.363 ± 0.434		2.547 ± 0.208		2.492 ± 0.661	
**Factor C: Sampling Time**									
		**Medium Milk (BBCH 75)**		**Early Dough (BBCH 83)**		**Ripening (BBCH 89)**			
*Fusarium* spp. (%)	**	2.20 ± 3.16	C	10.11 ± 6.60	B	14.74 ± 6.43	A		
*Aspergillus* spp. (%)	**	0.00 ± 0.00	B	0.32 ± 0.73	B	1.86 ± 2.77	A		
*Penicillium* spp. (%)	n.s.	1.00 ± 4.36		0.53 ± 1.43		1.47 ± 1.82			
Other fungi (%)	**	2.70 ± 3.18	C	53.68 ± 19.73	B	67.68 ± 11.36	A		
STC (µg/kg)	**	0.64 ± 1.39	B	1.93 ± 1.68	A	3.38 ± 3.84	A		
DON (µg/kg)	**	0.00 ± 0.00	B	30.20 ± 90.40	AB	61.78 ± 120.41	A		
TPC (mg GAE/g)	**	2.633 ± 0.574	A	2.238 ± 0.219	B	2.501 ± 0.343	A		
**Factors Interactions**									
		**A × B**		**A × C**		**B × C**		**A × B × C**	
*Fusarium* spp. (%)		n.s.		n.s		n.s.		n.s.	
*Aspergillus* spp. (%)		n.s.		**		**		n.s.	
*Penicillium* spp. (%)		n.s.		n.s		n.s.		n.s.	
Other fungi (%)		n.s.		**		n.s.		n.s.	
STC (µg/kg)		n.s.		**		n.s.		n.s.	
DON (µg/kg)		n.s.		*		n.s.		n.s.	
TPC (mg GAE/g)		**		**		**		**	

**Table 2:** Phenolic acid composition of the 4 rice varieties used for the study at different sampling times in 2018 and 2019. Data refer to mean data ± standard deviation; all experiments were conducted with three replicates.

		2018			2019		
Rice Variety	Phenolic Compound	Medium Milk (BBCH 75) mg/Kg	Early Dough (BBCH 83) mg/Kg	Ripening (BBCH 89) mg/Kg	Medium Milk (BBCH 75) mg/Kg	Early Dough (BBCH 83) mg/Kg	Ripening (BBCH 89) mg/Kg
**CL15**	Chlorogenic acid	9.69 ± 8.43	0.23 ± 0.05	<0.01	53.41 ± 21.30	109.45 ± 34.93	2.74 ± 0.27
	Caffeic acid	<0.01	<0.01	<0.01	<0.01	<0.01	<0.01
	Syringic acid	1.16 ± 0.56	0.43 ± 0.06	0.15 ± 0.10	<0.01	<0.01	<0.01
	4-hydroxybenzoic acid	11.83 ± 3.97	3.61 ± 0.27	2.13 ± 1.20	3.38 ± 0.19	1.75 ± 0.16	2.34 ± 0.23
	*p*-Coumaric acid	6.62 ± 1.71	1.39 ± 0.24	0.59 ± 0.17	2.31 ± 0.29	1.06 ± 0.27	1.24 ± 0.15
	Ferulic acid	4.13 ± 0.65	2.30 ± 0.02	2.59 ± 0.58	0.97 ± 0.40	0.80 ± 0.20	2.05 ± 0.31
	Protocatecuic acid	0.46 ± 0.01	0.27 ± 0.11	1.16 ± 0.99	0.14 ± 0.07	0.12 ± 0.04	0.92 ± 0.75
	Gallic acid	0.02 ± 0.02	<0.01	<0.01	0.28 ± 0.06	0.24 ± 0.02	0.27 ± 0.05
**CL26**	Chlorogenic acid	2.92 ± 0.59	0.20 ± 0.02	0.17 ± 0.03	152.35 ± 109.30	4.53 ± 4.54	15.88 ± 4.34
	Caffeic acid	<0.01	<0.01	<0.01	<0.01	<0.01	<0.01
	Syringic acid	0.64 ± 0.19	0.49 ± 0.05	<0.01	<0.01	<0.01	0.39 ± 0.33
	4-hydroxybenzoic acid	6.09 ± 0.72	4.82 ± 0.20	5.05 ± 0.31	20.02 ± 5.68	0.75 ± 0.90	2.94 ± 0.39
	*p*-Coumaric acid	2.38 ± 0.38	1.25 ± 0.23	0.81 ± 0.14	1.91 ± 0.32	0.27±0.23	1.34 ± 0.34
	Ferulic acid	2.16 ± 0.16	1.69 ± 0.02	2.53 ± 0.18	0.23 ± 0.17	0.29 ± 0.25	1.50 ± 0.09
	Protocatecuic acid	0.26 ± 0.01	<0.01	<0.01	0.14 ± 0.10	0.02 ± 0.02	0.38 ± 0.01
	Gallic acid	<0.01	<0.01	<0.01	0.26 ± 0.08	0.26 ± 0.07	0.17 ± 0.04
**SOLE CL**	Chlorogenic acid	1.76 ± 1.52	0.35 ± 0.24	0.78 ± 0.57	102.78 ± 57.19	82.79 ± 61.39	13.17 ± 3.62
	Caffeic acid	1.31 ± 0.99	<0.01	<0.01	<0.01	<0.01	<0.01
	Syringic acid	0.32 ± 0.05	<0.01	0.72 ± 0.02	<0.01	<0.01	<0.01
	4-hydroxybenzoic acid	9.90 ± 2.27	5.78 ± 1.24	3.03 ± 0.07	3.57 ± 0.12	1.71 ± 0.23	2.42 ± 0.27
	*p*-Coumaric acid	5.37 ± 1.93	1.98 ± 1.98	0.81 ± 0.14	2.49 ± 0.04	1.63 ± 0.38	1.39 ± 0.35
	Ferulic acid	3.93 ± 1.71	2.77 ± 0.99	5.92 ± 3.69	1.97 ± 1.03	1.88 ± 0.21	2.16 ± 0.35
	Protocatecuic acid	0.33 ± 0.04	0.20 ± 0.01	0.17 ± 0.10	0.18 ± 0.08	0.20 ± 0.08	0.44 ± 0.06
	Gallic acid	<0.01	0.08 ± 0.02	<0.01	0.19 ± 0.01	0.21 ± 0.01	0.17 ± 0.01
**TERRA CL**	Chlorogenic acid	14.46 ± 14.24	0.65 ± 0.11	0.05 ± 0.01	141.61 ± 31.00	75.74 ± 15.03	30.71 ± 8.03
	Caffeic acid	<0.01	<0.01	<0.01	<0.01	<0.01	<0.01
	Syringic acid	0.62 ± 0.14	<0.01	<0.01	<0.01	<0.01	0.29 ± 0.23
	4-hydroxybenzoic acid	21.82 ± 5.93	3.80 ± 0.12	4.41 ± 0.55	2.84 ± 0.16	1.33 ± 0.10	2.54 ± 0.12
	*p*-Coumaric acid	8.45 ± 0.42	1.17 ± 0.38	0.98 ± 0.05	1.43 ± 0.17	0.69 ± 0.13	0.88 ± 0.03
	Ferulic acid	4.07 ± 1.32	2.38 ± 0.71	3.77 ± 1.79	1.05 ± 0.15	0.88 ± 0.08	2.14 ± 0.47
	Protocatecuic acid	1.04 ± 0.04	0.61 ± 0.32	0.46 ± 0.01	0.28 ± 0.04	0.20 ± 0.11	0.59 ± 0.02
	Gallic acid	<0.01	0.01 ± 0.01	<0.01	0.23 ± 0.02	0.18 ± 0.01	0.18 ± 0.01

**Table 3:** Spearman’s correlation between phenolic acids. Correlation analysis was performed on 72 replicates (4 rice varieties × 3 sampling times × 3 replicates × 2 years). *: *p* ≤ 0.05; **: *p* ≤ 0.01.

Phenolic Acid	Chlorogenic Acid	Syringic Acid	4-hydroxybenzoic Acid	*p*-Coumaric Acid	Ferulic Acid	Protocatecuic Acid	Gallic Acid
Chlorogenic acid	1	−0.294 *	−0.253	0.182	−0.565 **	−0.198	0.626 **
Syringic acid		1	−0.350 **	0.190	0.447 **	0.159	−0.486 **
4-hydroxybenzoic acid			1	0.653 **	0.387 **	0.118	−0.481 **
*p*-Coumaric acid				1	0.270 *	0.153	−0.080
Ferulic acid					1	0.547 **	−0.664 **
Protocatecuic acid						1	−0.296 *
Gallic acid							1

**Table 4:** Spearman’s correlation between phenolic acids, main fungal Genera incidence and mycotoxin contamination. Correlation analysis was performed on 72 replicates (4 rice varieties × 3 sampling times × 3 replicates × 2 years). *: *p* ≤ 0.05; **: *p* ≤ 0.01.

Phenolic Acid	Fusarium spp. Incidence	Aspergillus spp. Incidence	Penicillium spp. Incidence	Total Fungi Incidence	STC	DON
Chlorogenic acid	−0.331 *	−0.285 *	−0.243	−0.141	−0.281	−0.576 **
Syringic acid	−0.156	0.171	0.003	−0.151	−0.230	−0.055
4-hydroxybenzoic acid	−0.348 **	−0.019	−0.054	−0.581 **	−0.314 *	−0.059
*p*-Coumaric acid	−0.497 **	−0.269 *	−0.140	−0.532 **	−0.427 **	−0.466 **
Ferulic acid	0.203	0.116	0.145	0.039	0.101	0.206
Protocatecuic acid	0.237	−0.026	0.355 **	0.156	−0.255	−0.021
Gallic acid	−0.068	−0.148	0.043	0.138	−0.102	−0.183

## Supplementary Materials

The following are available online at https://www.mdpi.com/2072-6651/12/5/341/s1, Table S1: Mean daily temperature (°C) and rainfall (mm) registered during the rice growing season in Castello d’Agogna (PV) in 2018 and 2019.
