# Supplementary material for: Monitoring Phenolic Compounds in Rice during the Growing Season in Relation to Fungal and Mycotoxin Contamination
Source: Toxins (Basel). 2020 May 22;12(5):341. doi: 10.3390/toxins12050341 (PMC7291125; doi:10.3390/toxins12050341)
Supplement: Supplementary file 1 [file toxins-12-00341-s001.pdf]

Supplementary Materials: Monitoring Phenolic Compounds in Rice During the Growing Season in Relation to Fungal and Mycotoxin Contamination

Paola Giorni, Silvia Rastelli, Sofia Fregonara and Terenzio Bertuzzi

Table S1. Mean daily temperature (°C) and rainfall (mm) registered during the rice growing season in Castello d'Agogna (PV) in 2018 and 2019.

**Mean Daily Temperature (°C)**							
	**March**	**April**	**May**	**June**	**July**	**August**	**September**
**Year 2018**	7.0	15.9	19.7	24.3	26.0	25.4	21.2
**Year 2019**	10.0	13.8	16.3	25.4	26.2	25.0	19.9
**Rainfall (mm)**							
	**March**	**April**	**May**	**June**	**July**	**August**	**September**
**Year 2018**	90.8	87.8	111.4	16.2	74.4	27.2	46.6
**Year 2019**	3.6	82.8	68.2	2.0	50.8	45.6	28.4
